# PReS-FINAL-2345: Frequency of radiographic damage and progression in individual joints in children with juvenile idiopathic arthritis

**DOI:** 10.1186/1546-0096-11-S2-P335

**Published:** 2013-12-05

**Authors:** G Giancane, S Pederzoli, X Norambuena, M Ioseliani, J Sato, MC Gallo, G Negro, N Ruperto, A Martini, A Ravelli

**Affiliations:** 1 Istituto Giannina Gaslini, Genova, Italy

## Introduction

Juvenile idiopathic arthritis (JIA) may lead to permanent damage of the articular cartilage and bone. Because the prevention of irreversible joint changes is a key objective in the long-term management of chronic arthritis, evaluation of radiographic joint damage represents an important clinical tool for assessing disease severity and progression and for monitoring the effectiveness of therapeutic interventions. Although newer imaging techniques, such as MRI and ultrasound, allow earlier detection of bone and cartilage changes, conventional radiography remains the gold standard for the demonstration of structural joint lesions in patients with JIA. Importantly, mapping the prevalence and location of structural abnormalities in different sites on conventional radiology may provide useful information to guide future investigations with MRI and ultrasound.

## Objectives

To evaluate the presence and progression of radiographic joint damage, as assessed with the adapted Sharp-van der Heijde (aSH) score, in individual joints in the hand and wrist in patients with JIA, and to compare progression of damage among different JIA categories.

## Methods

A total of 372 radiographs of both wrists and hands obtained at first observation and at last follow-up visit (after 1 to 10 years) in 186 children with polyarticular-course JIA were evaluated. All radiographs were scored using the aSH scoring system by 2 independent readers. Radiographic assessment included evaluation of joint space narrowing (JSN) and erosions on baseline and last follow-up radiographs and of progression of radiographic changes from baseline to last follow-up radiographs.

## Results

Both JSN and erosions occurred in all aSH areas. Overall, radiographic damage and progression were more common in the wrist and less common in metacarpophalangeal joints. The hamate and capitate areas appeared particularly vulnerable to cartilage loss. Erosions were identified most frequently in the hamate and capitate bones as well as in the 2^nd ^and 3^rd ^metacarpal bases. Patients with extended oligoarthritis were distinctly less susceptible to JSN in hand joints, whereas patients with polyarthritis showed a greater tendency to developing erosions in hand joints.

## Conclusion

Radiographic joint damage and progression in our patients with JIA were seen most commonly in the wrist and less commonly in MCP joints. The frequency and localization of structural abnormalities differed markedly across disease categories.

## Disclosure of interest

None declared.

